# Salivaomics: A Revolutionary Non-invasive Approach for Oral Cancer Detection

**DOI:** 10.7759/cureus.74381

**Published:** 2024-11-25

**Authors:** Girish Suragimath, Satish Patil, Disha G Suragimath, Ashwinirani SR

**Affiliations:** 1 Periodontology, Krishna Vishwa Vidyapeeth (Deemed to be University), Karad, IND; 2 Microbiology, Krishna Vishwa Vidyapeeth (Deemed to be University), Karad, IND; 3 General Medicine, Krishna Vishwa Vidyapeeth (Deemed to be University), Karad, IND; 4 Oral Medicine and Radiology, Krishna Vishwa Vidyapeeth (Deemed to be University), Karad, IND

**Keywords:** biomarkers, carcinogenesis, oral cancer, oral potentially malignant disorder, saliva, salivaomics

## Abstract

Salivaomics has emerged as a ground-breaking field in the detection and management of oral cancer (OC), offering a non-invasive, efficient, and patient-friendly alternative to traditional diagnostic methods. This innovative approach leverages the comprehensive molecular insights provided by genomics, transcriptomics, proteomics, metabolomics, and microbiomics. The potential of salivaomics lies in its ability to enable early detection, predict malignant transformation, and monitor treatment outcomes and disease recurrence. Advancing salivary diagnostics necessitates the standardization of saliva collection and processing protocols, identification and validation of robust biomarkers, and development of cutting-edge detection technologies. A single biomarker is unlikely to fulfill all diagnostic requirements; thus, research should focus on developing a panel of biomolecules to enhance diagnostic accuracy and management of OC. Salivaomics stands at the forefront of non-invasive diagnostic methods, with the promise to revolutionize early detection and management of OC. Future research directions should emphasize the integration of multi-omics data for superior biomarker discovery, the development of portable and cost-effective point-of-care devices, and the fostering of interdisciplinary collaborations to drive innovation. Overcoming these challenges will facilitate the translation of salivaomics into routine clinical practice, significantly improving early diagnosis, treatment, and prognosis of OC. This review provides a comprehensive overview of salivaomics, detailing the use of saliva as a diagnostic fluid. It covers saliva collection, preparation, transportation, storage methods, and various analytical techniques. Additionally, the review discusses the current challenges and future directions of this transformative technology, emphasizing its potential to enhance clinical outcomes in OC.

## Introduction and background

Salivaomics is the scientific field of measuring biological molecules in the saliva for diagnosis and prognosis of disease processes in the body. Salivaomics is an emerging field, which focuses on saliva-based techniques for the early diagnosis and measurement of treatment outcomes of diseases [1]. Salivaomics holds the potential to offer clinicians a robust alternative for making informed clinical decisions and improving treatment outcomes. Salivaomics technology can further be subdivided depending upon the molecule assessed as salivary genomics, transcriptomics, proteomics, epigenomics, metabolomics, and microbiomics [2].

Oral cancers (OCs) also known as mouth cancers, encompass malignancies that develop in any part of the oral cavity like lips, tongue, cheeks, floor of the mouth, hard and soft palates, sinuses, and throat. OCs are primarily termed squamous cell carcinomas, as 90% of them histologically originate from the oral squamous cells [3]. OCs are ranked among the 10 most common cancers globally, characterized by delayed clinical detection and poor prognosis. The disease lacks specific biomarkers and often involves expensive therapeutic alternatives. Despite advances in research and therapy, the survival rates for OC have not improved significantly in recent years, posing a major challenge for biomedical science [4]. Early detection and appropriate treatment can reduce the risk to a great extent, thereby reducing the burden of OC [5].

Traditional diagnostic methods like biopsies and imaging are invasive, costly, and not always conducive to early detection of OC [6]. Saliva collection and analysis are a promising alternative as it is easy and non-invasive and contain rich diagnostic biomarkers, which are in direct contact with OC lesions making it a potentially sensitive screening tool for early detection. This review aims to provide a comprehensive overview of the use of saliva as a diagnostic fluid; enlightens in detail about saliva collection, preparation, transportation, and storage methods; and explains different saliva analytical techniques of salivaomics, present state, current challenges, and future direction of this innovative technology.

## Review

Methodology

Study Design

This comprehensive review was performed as per the Preferred Reporting Items for Systematic Reviews and Meta-Analysis (PRISMA) guidelines. The research questions were salivaomics, saliva collection, preparation, transportation, storage methods, and various analytical techniques. Additionally, we searched the current challenges and future directions of saliva as a diagnostic fluid for the detection of OC.

Inclusion and Exclusion Criteria

We included full-text articles in English published between January 1974 and September 2024. Research studies on salivaomics technologies, reviews, and online literature describing saliva as a diagnostic fluid for the detection of OC and oral potentially malignant disorders (OPMDs) were considered. We excluded editorials, opinions, letters to the editor, studies using gingival crevicular fluid, biopsies, blood samples, and plasma samples for OC detection.

Search Strategy

The search strategy involved using several online databases like PubMed, Google, Scopus, Cochrane Library, and Web of Science. The Medical Subject Headings (MeSH) search terms used included “oral cancer”, “mouth neoplasms”, “head and neck cancer”, “precancerous conditions”, “saliva”, “biomarker”, and “diagnosis”. The other terms used were saliva collection, preparation, transportation, storage methods, various analytical techniques, current challenges, and future directions. A manual search was also conducted combining “AND” or “OR” operators.

Data Extraction

**Figure 1:**
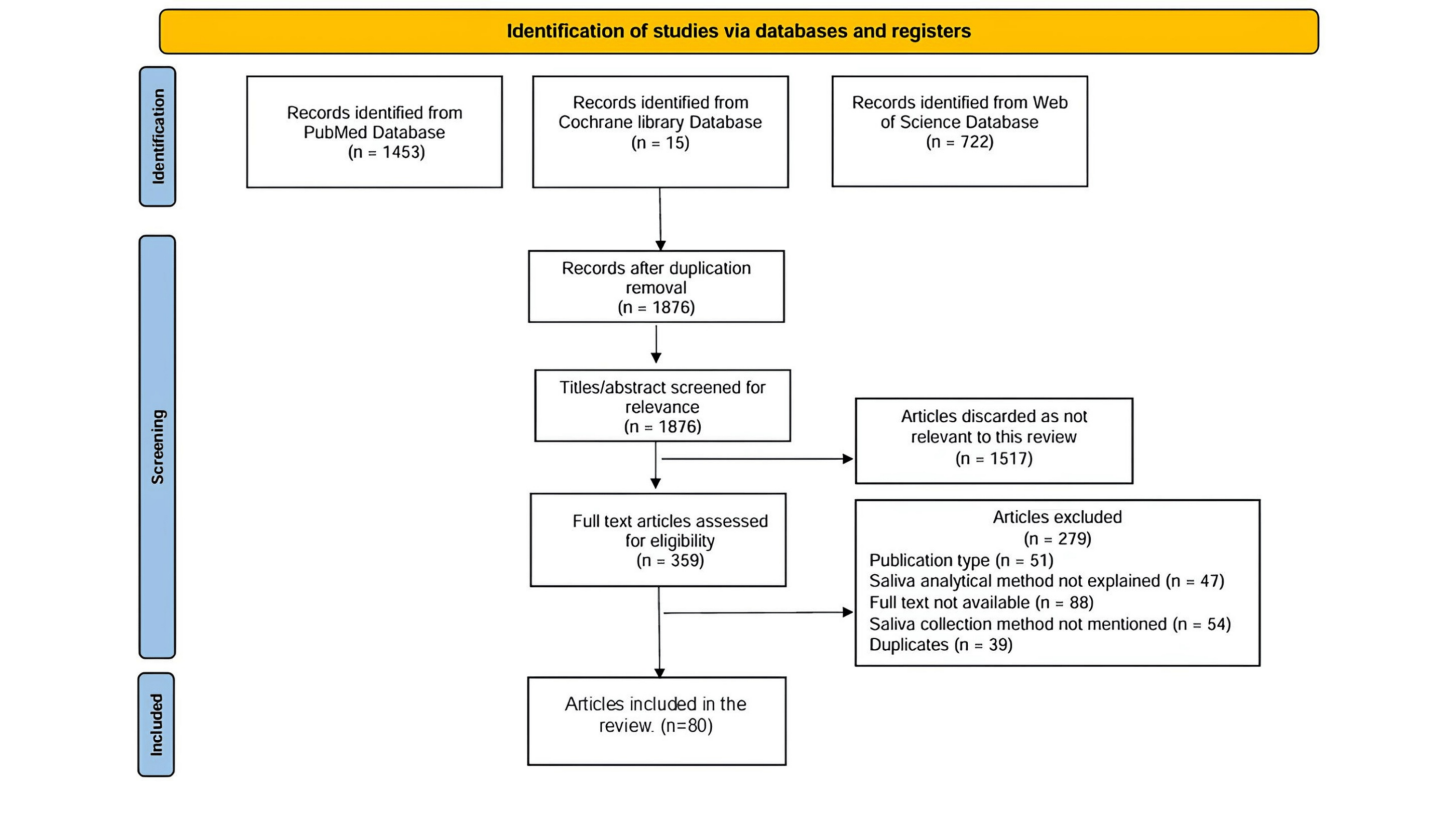
PRISMA flowchart of selected articles for the review. PRISMA: Preferred Reporting Items for Systematic Reviews and Meta-Analysis

Data extraction involved collecting relevant information like objectives, methods, results, and conclusions, from the extracted articles. A total of 359 articles were recognized after the literature search, and 80 pertinent articles were selected to be explained and discussed in this review (Figure 1).

Discussion

Advantages and Drawbacks of Saliva as a Diagnostic Fluid

The advantages and drawbacks of using saliva as a biomarker are listed in Table 1 [7-10].

**Table 1:** Advantages and drawbacks of using saliva as a diagnostic fluid.

Advantages	Drawbacks
Non-invasive collection: simple and painless, enhancing patient compliance and comfort	The levels of some biomarkers in the saliva vary and are not as accurate and reliable depending on the collection methods.
Cost-effective: less expensive, making it accessible for widespread screening programs	The method of saliva stimulation, collection, salivary pH, and flow rate may alter the concentration of salivary biomarkers.
Easy storage and transport: samples can be stored and transported easily without the need for specialized conditions	The quantity and quality of saliva vary from individual to individual and during different time periods of the day and different environmental conditions.
Frequent monitoring: easy non-invasive collection allows for regular monitoring of disease progression and response to treatment	Salivary contamination can occur depending on the food/beverage consumed, gingival crevicular fluid, microbial plaque, bleeding from the gums, and oral wounds.
Safe and viable: saliva collection is safe for health workers, poses minimal risk of cross-contamination, requires less manipulation, and allows for home collection and screening	Systemic diseases, medications, and radiation therapy affect the salivary gland function altering the quantity and quality of saliva collected.
Real-time diagnostic: saliva analysis has real-time diagnostic value and can be used for real-time applications	Proteolytic enzyme derivatives from the host and oral microbes in saliva collected may alter the state of diagnostic markers, affecting the final analysis.

Saliva Collection, Preparation, Transportation, and Storage Methods

Saliva collection, preparation, transportation, and storage for biomarker analysis can be achieved by different methods [11-15]. Saliva secretion and collection can be in two ways: stimulated with the use of salivary stimulants like paraffin wax or citric acid and unstimulated. Saliva can be collected and investigated by four types of collection methods, i.e., whole mouth saliva, submandibular saliva, parotid saliva, and sublingual saliva.

Saliva collection methods:* *Patients tilt their heads down and let saliva accumulate in their mouth without swallowing and expectorate into the saliva-carrying unit in a passive drooling method, spitting saliva into the saliva-carrying unit continuously or with intervals of passive drooling [12,13]. A guiding tool for self-collection can also be used with the spitting method. Non-coated, polypropylene-covered, or citric-acid-coated cotton rolls are placed inside the mouth to absorb saliva for further analysis. The sorbette kit (Salimetrics® collection kit, Carlsbad, CA, US) has three sorbettes (cotton pads on a stick) to be placed under the patient’s tongue for collection and analysis of saliva. An ophthalmic sponge (Merocel®, Medtronic, Minneapolis, MN, US) or swab (microFLOQ®, Copan, Italy) is placed under the tongue for 30 seconds for saliva collection. Suction methods using needleless syringes, plastic catheters, (micro) pipettes, or plastic droppers are favored when procuring saliva samples from infants, children, or patients with special needs [13].

Saliva preparation, transportation, and storage: Saliva collected should be transported immediately to the laboratory at room temperature or in an icebox at 4° to 6° temperature [12,14]. Salivary samples are prepared and stored by different methods mentioned here: centrifugation of the saliva sample at 1,500 rpm for two minutes to remove cell debris and storage at -20°C or -70°C to 80°C until assay, storage of the salivary sample at 4°C without centrifugation and assay within 24 hours, storage of the salivary sample at 37°C or room temperature without centrifugation and assay within 24 hours, centrifugation of the salivary sample immediately at 1,500 rpm for two minutes and assay without storage, and analysis of the salivary sample immediately without centrifugation or storage. Analysis of the saliva samples immediately after collection without centrifugation or storage is the best method for the analysis of the biomarkers [12,15].

Saliva Sampling Time

The timing of saliva collection can significantly impact the reliability and validity of the biomarker measured [16-19]. The optimal sampling time varies depending on the analytes being measured and the type of biomarker assessed. Factors influencing saliva sampling time are circadian rhythms (diurnal variations), dietary intake, oral hygiene, physical activity, and stress and emotional state. Multiple samples throughout the day may be needed to map diurnal variations for hormones like cortisol and melatonin. Sampling in a resting state, typically mid-morning, can provide a baseline measurement for enzymes. Mid-morning sampling is often recommended for saliva collection for protein (immunoglobulins) analysis. Fasting samples or standardized postprandial times are suited for metabolites like glucose and lactate estimation. The detection of OC metabolites in saliva is less influenced by circadian rhythms but can be affected by daily activities such as eating, drinking, and oral hygiene practices. Salivary sample collection between the times of 7:30 AM to 9:00 AM, 10:30 AM to 11:00 AM, and 02:00 PM to 08:00 PM ensures that the samples are collected during a period of relatively stable metabolic activity [18].

Best Practices for Saliva Sampling

Adhering to standardized protocols for saliva collection, handling, and storage is essential for minimizing variability and ensuring reproducibility of results [9]. Maintaining consistent timing at the same time of day for saliva collection for longitudinal studies or repeated measures reduces the impact of diurnal variations. Providing clear instructions to participants regarding fasting, eating, drinking, oral hygiene, and avoiding certain activities before sampling yields better results. The use of appropriate collection techniques/devices depending on the analytes of interest is necessary. Passive drool is often preferred for hormonal analysis, while swabs may be more convenient for field studies. Proper transport and storage of samples will prevent degradation, and saliva samples are usually stored at -20°C or lower until analysis.

Salivary Analytical Technologies

The techniques used for biomarker identification and profiling vary depending on the sample preparation, operating principle, and interpretation of results [19-24]. Polymerase chain reaction (PCR) is used for detecting specific deoxyribonucleic acid (DNA) and ribonucleic acid (RNA) sequences. Enzyme-linked immunosorbent assay (ELISA) is utilized for quantifying proteins and other biomolecules. Mass spectrometry (MS) is useful for the comprehensive profiling of proteins and metabolites. Next-generation sequencing provides detailed genetic information from saliva samples. Liquid chromatography-MS (LC-MS) is a powerful analytical technique for initial biomarker identification [22]. High-performance LC (HPLC) and gas chromatography (GC) are analytical chemistry methods for analyzing compounds that vaporize without decomposition. Nuclear magnetic resonance (NMR) is used in metabolomics biomarker identification and is robust and reproducible [23]. Capillary electrophoresis-MS (CE-MS) is a separation technique based on the movement of ions under electrophoretic and/or electro-osmotic forces, produced by the application of an electric field, combined with a mass spectrometer [24]. Advances in analytical techniques have led to a significant increase in the identification of new biomarkers. These new technologies for identifying biomarkers are highly sophisticated, requiring a perfect environment for analysis, expensive sensitive equipment, and highly qualified personnel to carry out the analysis. All these requirements may not be feasible in all the general laboratory setups.

Clinical Applications of Salivary Analysis

Salivary diagnostics can help in the early detection of OC, improving prognosis and survival rates. Regular saliva tests can help monitor the progression of the disease and can be used to assess the effectiveness of OC treatment. Genetic and molecular profiling of saliva can help in assessing the risk of developing OC from OPMDs. Saliva biomarkers can also be used to predict the recurrence of OC after successful treatment for long-term follow-up of patients [25].

Biomarkers in Saliva

Saliva contains a variety of biomarkers that can be indicative of OC, and salivaomics leverages advanced technologies to analyze the molecular constituents of saliva. Genetic biomarkers involve studying the DNA in saliva, i.e., genomics, to identify mutations and genetic predispositions. Genetic alterations, such as mutations in the TP53 gene or human papillomavirus DNA, can be detected in the salivary DNA of OC patients. Transcriptomics involves examining RNA profiles to detect changes in gene expression associated with OC. Dysregulated microRNAs (miRs), such as miR-21, miR-184, miR-125a, miR-200a, miR-let-7-5p, miR-412-3p, miR-512-3p, miR-302-3p, miR-517-3p, miR-30c-5p, miR-SAT, miR-OAZ, miR-H3F3A, and miR-24-3p, have all shown significant differences among OPMD and OC patients compared to control groups [26]. Protein biomarkers like cytokines, enzymes, and growth factors have been assessed. The salivary expression of cytokines, like interleukin (IL)-4, IL-6, IL-10, IL-13, IL-1β, IL-1RA, IL-17A, and IL-17F; interferon-gamma (IFN-γ); tumor necrosis factor-alpha (TNF-α); and growth factors like hepatocyte growth factor (HGF), C-reactive protein (CRP), and vascular endothelial growth factor (VEGF), have been studied. Increased activity of enzymes like matrix metalloproteinases (MMPs) can indicate tumor invasion. Salivary MMP-9 and MMP-2 are extensively studied; other enzymes assessed are lactate dehydrogenase (LDH), aldo-keto reductase family 1 member B10 (AKR1B10), L-fructose, cathepsin V, and kallikrein. Changes in the levels of certain metabolites, such as polyamines, ornithine, ohydroxyvenzonate, tumor-specific antigen (TSA), and ribose 5-phosphate (R5P), are associated with OC. Other biomarkers like angiotensin (ANG), ANG2, nucleolar complex protein 1 (NUS1), transgelin, reticulocalbin 1 (RCN1), karyopherin alpha 2 (KPNA2), galectin-3-binding protein (LGALS3BP), vitamin C, L-fructose, cytokeratin fragment 21-1 (CYFRA 21-1), β2-microglobulin, cathepsin B, fibrin split products A (FSA), and para-benzenesulfonic acid (PBSA) have shown significant increases in patients with OC. Alterations in the composition of oral microbiota, with an increase in potentially pathogenic organisms including higher levels of Fusobacterium nucleatum, have been observed in OC patients [27]. The treatment outcomes can also be measured with microbiome biomarkers to calculate response to treatment with the use of Gemella species and Leptotrichia species [28].

The biomarkers for OC can be divided into enzymes, glycoproteins, cytokines, miRs, metabolites, treatment prognostic markers of OC, prediction of malignant transformation, and other salivary biomarkers.

Enzymes:Enzymes like MMP, LDH, AKR1B10, L-fructose, cathepsin V, and kallikrein have been found in higher concentrations in OC. A study by Kochurova and Nikolenko showed that salivary MMP-9 and MMP-2 had statistically significant concentrations in patients with OPMD compared to healthy controls [29]. Significant levels of salivary MMP-9 were observed in patients with OC compared to OPMD patients in another study [30]. A study by Ghallab and Shaker proved that the salivary levels of MMP-9 can be utilized to differentiate OPMD and OC with 100% specificity and sensitivity [31]. Additional MMPs have also been studied and found to be altered in OC patients [32,33]. LDH is one of the most studied enzymes, and a substantial increase in LDH levels is found in OC and premalignant lesions compared to the control groups [34,35]. The salivary levels of AKR1B10 were least in healthy controls (25.0.1 ± 32.96 pg/mL) and significantly greater in OC patients (646.47 ± 402.43 pg/mL), and OC patients with values more than 646 pg/mL showed lower survival rates [36]. The analysis of combination enzymes and proteases like kallikrein 5, cathepsin V, and A disintegrin and a metalloprotease 9 (ADAM9) improved the diagnostic accuracy of OC [37].

Glycoproteins:Saliva consists of serous and mucous glycoproteins like cluster of differentiation 44 (CD44) and carcinoembryonic antigen (CEA), which have been found in higher proportion in OC patients. A significantly raised level of CEA has been observed in the salivary samples of patients with OC [38,39]. The variants of CD44 mainly CD44v6 have a probable role in the early stages of OC, and CD44v6 and CD44v10 were elevated in histopathological aggressive OC [40].

Cytokines:Salivary expression of diverse cytokines has been studied comprising IL-1β, IL-1RA, IL-4, IL-6, IL-10, IL-13, IL-17A, IL-17F, TNF-α, IFN-γ, HGF, CRP, and VEGF [41,42]. IL-8 has been the most studied and found to be elevated at a significant level [43-45]. A study reported that both IL-1β and IL-8 are effective salivary biomarkers for the diagnosis of OC at all stages, specifically in stages III and IV OC and in the postoperative phase [46]. A study showed overexpression of IL-17A and IL-17F in salivary samples with advanced stages of OC and higher lymph node involvement [47]. The salivary concentration of TNF-α has been found to be significantly greater in OC patients [43,44] and OPMD subjects than the control subjects [48]. Other cytokines like HGF and VEGF have also been found at higher concentrations in OC patients [49].

miRs: Various studies have measured concentrations of diverse miRs in salivary samples of patients suffering from OC and OPMD. miRs like miR-let-7-5p, miR-21, miR-184, miR-30c-5p, miR-302-3p, miR-412-3p, miR-512-3p, miR-517-3p, miR-SAT, miR-OAZ, miR-H3F3A, and miR-24-3p have revealed significant differences among patients with OPMD and OC when equated with control subjects [50-52]. Rajaram et al. cautioned the use of salivary RNA levels for OC diagnosis, as the values of the same may be altered due to periodontal disease and status [53].

Metabolites:Ishikawa et al. explored three metabolites, i.e., ohydroxyvenzonate, ornithine, and R5P, to find the difference in their concentration in patients with oral dysplasia and OC, and found variations in the concentration of these metabolites [54]. TSA concentrations in saliva were compared with grades of cancer cell differentiation and found a significant increase in TSA levels with a higher degree of cell differentiation [53,55]. Proline and glycine are also significantly raised in the salivary samples of patients suffering from OC [56].

Other salivary biomarkers:The other salivary biomarkers studied include ANG, NUS1, RCN1, transgelin, FSA, PBSA, KPNA2, LGALS3BP, vitamin C, L-fructose, CYFRA 21-1, β2-microglobulin, and cathepsin B. Biomarkers ANG, ANG2, NUS1, transgelin, and RCN1 are found to be significantly raised in OC [49,57-59]. A study by Azeem et al. analyzed the levels of PBSA and FSA in OC patients with tobacco use, non-cancer patients with tobacco use, and healthy subjects without tobacco use and found significantly raised levels of both markers among tobacco users and OC patients [60]. The salivary concentrations of markers LGALS3BP and KPNA2 are raised in OC patients [46,61]. Different studies have found altered levels of vitamin C, L-fructose, CYFRA 21-1, β2-microglobulin, and cathepsin B and expression gene coding of diverse proteins among OC patients [62-70].

Treatment prognostic markers of OC:The outcome prediction of OC has been studied with different biomarkers like CYFRA-21, IL-5, miR-139-5p, and epidermal growth factor receptor (EGFR) [55,71-74]. Salivary miR-139-5p has shown to be a predictive marker after surgical treatment after tumor excision [72]. The biomarker CYFRA 21-1 levels in saliva have been shown to be related to the recurrence of OC [73]. Poor survival of OC patients has been observed with raised levels of EGFR in saliva [74]. A study conducted by Val et al. found that the levels of IL-5 increased after the first surgery, subsequently reduced when a second lesion appeared, and then increased again in saliva postremoval of the second lesion [71].

Prediction of malignant transformation of OPMDs:Few biomarkers in saliva have been used to foretell the malignant change of the OPMDs like miRNA-2, miRNA-184, malondialdehyde (MDA), glutathione peroxidase (GPx), TNF-α, and alpha-fetoprotein (AFP). A study by Zahran et al. found raised levels of miRNA-21 and miRNA-184 in the saliva of patients with OPMDs and OC compared to healthy controls [52]. Sabarathinam et al. found levels of salivary GPx, MDA, TNF-α, and AFP progressively raised during the transformation of lesions from OPMDs to OC [75]. Hung et al. proved that levels of miRNA-31 in saliva increased in OPMDs and oral lichen planus during the malignant transformation [76].

Current Challenges

Several challenges need to be addressed, and future directions must be considered to improve the efficacy and reliability of using salivaomics for the detection of OC [77-80]. Standardization of saliva collection, storage, and processing is a must, as variability in saliva collection methods (unstimulated vs. stimulated) and storage conditions can affect the consistency and reliability of the results. Different processing techniques can lead to variations in the detection of biomarkers. Identifying biomarkers that are both highly specific and sensitive to OC is challenging. Extensive clinical validation is required to confirm the diagnostic value of identified biomarkers. Interindividual variability such as age, gender, diet, oral hygiene, and systemic health can influence salivary composition, leading to variability in biomarker levels. There is a need for advanced, sensitive, and cost-effective technologies to detect low-abundance biomarkers. Complex data analysis methods are required to interpret the results accurately. Regulatory approval processes can be lengthy and complex to develop new technologies for biomarker identification. Gaining acceptance from clinicians and integrating salivary diagnostics into routine clinical practice can be challenging.

Future Directions

Future directions should point to advancements in biomarker research by integrating genomics, proteomics, metabolomics, and transcriptomics to identify robust biomarkers; continuous discovery of new biomarkers and validation through large-scale studies; implementation of automated systems to minimize human error and improve reproducibility; utilization of advanced technologies such as nanotechnology, microfluidics, and biosensors for more sensitive and specific detection; and development of portable, user-friendly devices for real-time salivary diagnostics. Artificial intelligence (AI) including machine learning (ML) and deep learning (DL) strategies will drastically improve salivaomics and its applications, mainly with large datasets. The use of mobile-based sensor technology can improve OC screening services even in remote areas. Salivary biomarkers could be used to stratify patients based on their risk of developing OC and tailor preventive strategies accordingly. Salivary diagnostics can be employed to monitor treatment response and detect recurrence. Fostering collaboration between researchers, clinicians, and industry is essential to accelerate the development and implementation of salivary diagnostics. Integrating salivary diagnostics into public health screening programs can improve early detection and reduce the burden of OC. Working with regulatory bodies to develop clear guidelines and streamline the approval process for salivary diagnostic tests is important. Ensuring the highest ethical standards are maintained in the collection, use, and storage of salivary samples is paramount. By addressing these challenges and focusing on these future directions, the use of salivary samples for OC detection can become a reliable, non-invasive, and widely adopted diagnostic tool.

## Conclusions

Salivaomics represents a transformative approach in the field of OC detection, offering a non-invasive, efficient, and patient-friendly alternative to traditional diagnostic methods. By encompassing genomics, transcriptomics, proteomics, metabolomics, and microbiomics, salivaomics provides a comprehensive view of the molecular landscape associated with OC. It is poised to significantly impact OC therapy through early detection, prediction of malignant transformation, and the measurement of treatment outcomes and disease recurrence. To ensure the reliability and reproducibility of salivary diagnostics, standardization of saliva collection and processing protocols is essential. Researchers generally agree that a single biomarker cannot meet all the requirements of an ideal diagnostic tool. Therefore, research should focus on developing a panel of biomolecules to achieve the accuracy needed for the diagnosis and management of OC. Continued research and technological advancements are crucial in overcoming existing challenges and translating salivaomics into routine clinical practice. This will ultimately enhance early diagnosis, treatment, and prognosis of OC. Future directions in salivaomics research should prioritize the integration of multi-omics data to improve biomarker discovery and the development of portable and cost-effective point-of-care devices for the effective management of OC.
